# Different Roles for Honey Bee Mushroom Bodies and Central Complex in Visual Learning of Colored Lights in an Aversive Conditioning Assay

**DOI:** 10.3389/fnbeh.2017.00098

**Published:** 2017-05-30

**Authors:** Jenny A. Plath, Brian V. Entler, Nicholas H. Kirkerud, Ulrike Schlegel, C. Giovanni Galizia, Andrew B. Barron

**Affiliations:** ^1^Department of Biological Sciences, Macquarie UniversitySydney, NSW, Australia; ^2^Department of Biology, University of KonstanzKonstanz, Germany; ^3^Department of Biology, University of ScrantonScranton, PA, United States; ^4^International Max-Planck Research School for Organismal Biology, University of KonstanzKonstanz, Germany; ^5^Department of Biosciences, University of OsloOslo, Norway

**Keywords:** visual learning, operant learning, mushroom bodies, central complex, honey bees, procaine

## Abstract

The honey bee is an excellent visual learner, but we know little about how and why it performs so well, or how visual information is learned by the bee brain. Here we examined the different roles of two key integrative regions of the brain in visual learning: the mushroom bodies and the central complex. We tested bees' learning performance in a new assay of color learning that used electric shock as punishment. In this assay a light field was paired with electric shock. The other half of the conditioning chamber was illuminated with light of a different wavelength and not paired with shocks. The unrestrained bee could run away from the light stimulus and thereby associate one wavelength with punishment, and the other with safety. We compared learning performance of bees in which either the central complex or mushroom bodies had been transiently inactivated by microinjection of the reversible anesthetic procaine. Control bees learned to escape the shock-paired light field and to spend more time in the safe light field after a few trials. When ventral lobe neurons of the mushroom bodies were silenced, bees were no longer able to associate one light field with shock. By contrast, silencing of one collar region of the mushroom body calyx did not alter behavior in the learning assay in comparison to control treatment. Bees with silenced central complex neurons did not leave the shock-paired light field in the middle trials of training, even after a few seconds of being shocked. We discussed how mushroom bodies and the central complex both contribute to aversive visual learning with an operant component.

## Introduction

Learning of a predictive relationship between a stimulus or an action and a certain outcome is essential for an animal's survival. Honey bees are excellent learners, quickly forming association between stimuli of different sensory modalities and meaningful appetitive and aversive stimuli (Giurfa, 2007). Over the past decades, research has been dedicated to uncover the neural mechanisms and processes underlying learning in bees, and honey bees have been established as a powerful model to investigate learning and memory (Menzel, 1999, 2001, 2012; Giurfa, 2003, 2007). Learning assays are typically performed with free-flying bees as well as harnessed bees (Menzel, 1999, 2001; Giurfa, 2003, 2007; Menzel, 2012). Free-flying bees readily learn olfactory as well as visual stimuli. Appetitive learning and memory dynamics have been studied extensively using odors and colors or shapes paired with sucrose rewards.

Harnessed bees have been used in the proboscis extension response (PER) assay, in which the conditioned stimulus (CS) is paired with a sucrose reward (unconditioned stimulus: US) which leads to an extension of the proboscis (Bitterman et al., 1983; Felsenberg et al., 2011; Giurfa and Sandoz, 2012). Olfactory conditioning is easily studied with this assay since 50–60% of the trained bees already respond to an odor after one CS-US pairing (Bitterman et al., 1983; Felsenberg et al., 2011). It has proven difficult, however, to achieve successful conditioning of color stimuli with rewards or punishment in harnessed honey bees. Differential conditioning with a reward-paired color stimulus and a non-rewarded color stimulus resulted in moderate learning rates when the antennae were ablated (Kuwabara, 1957; Hori et al., 2006, 2007; Niggebrugge et al., 2009), when the bee was able to turn her head easily (Dobrin and Fahrbach, 2012) or when the color stimulus was combined with movement (Balamurali et al., 2015). Colored light, however, has been used successfully as a context for olfactory learning in PER when presented as an occasion-setter (Mota et al., 2011) or in a reinstatement paradigm (Plath et al., 2012). The difficulty in establishing robust visual learning in the PER assay has inhibited functional analyses of roles of different brain regions in visual learning in bees.

Here we used a recently developed aversive visual conditioning assay: the Automated Performance Index System (APIS) (Kirkerud et al., 2017) to analyze the roles of central processing regions of the bee brain in visual learning. This system was an adapted version of the one used for aversive olfactory conditioning (Kirkerud et al., 2013; Schott et al., 2015; Wehmann et al., 2015). In the APIS assay bees are able to move freely in a conditioning chamber, which is equipped with LEDs to provide visual stimuli of different wavelengths and intensities. Visual stimuli can be paired with low voltage electric shocks. Tracking of the animal's position is fully automated thanks to infrared sensors in the chamber. The chamber can be used to investigate differential learning presenting light in half of the chamber and light with different properties in the other half. One light field is paired with electric shock, so that the bee needs to cross over to the other half of the chamber to avoid being shocked. The assay has been extensively tested with different light stimuli including light of different wavelengths and intensities (Kirkerud et al., 2017). Bees easily learn to associate 465 nm light (blue for humans) and 590 nm light (yellow for humans) but not 525 nm light (green for humans; in the following, we use the human colors instead of the wavelengths for simplicity) with the aversive shock stimulus. In this study, we paired blue light with shocks in one half of the chamber and illuminated the “safe” part of the chamber with green light. Bees can be treated pharmacologically and then their behavior can be assessed in the APIS chamber. Here, we investigated the role the mushroom bodies (MBs) and the central complex (CX) in visual learning.

MBs and the CX are considered the main integrative centers in the insect brain, and both regions could be involved in learning an appropriate behavioral response to a visual stimulus. We investigated the behavioral consequence of silencing of the input region of the MBs, the collar region in the mushroom body calyces (MBC), and the vertical lobes (VL) as the output region of the MBs. The collar region receives direct visual input from the lobula and medulla in honey bees (Ehmer and Gronenberg, 2002; Gronenberg and Lopez-Riquelme, 2004). A recent study has found two types of Kenyon cells in the fruit fly MBC that respond to either light intensity or wavelength (color) information relayed from the optic neuropils (Vogt et al., 2016). Interestingly, in flies both types of neurons are required for learning and memory in an aversive differential conditioning, either testing different intensities or different wavelengths. The output of the collar region in the mushroom bodies terminates in an inner layer of the vertical lobes in honey bees (Strausfeld, 2002). It has been repeatedly shown that the vertical lobes play a crucial role for different forms of olfactory learning and memory formation in honey bees (Menzel, 1999, 2012) and fruit flies (Heisenberg, 2003; Keene and Waddell, 2007; Busto et al., 2010; Davis, 2011), but visual learning has only been investigated sparsley so far.

The CX comprises a group of unpaired neuropils in the center of the insect brain. One important role of the CX is generation of motor outputs according to processed internal and external stimuli (Pfeiffer and Homberg, 2014; Plath and Barron, 2015). The CX is essential for the initiation and termination of walking, turning and climbing behavior in fruit flies (Strauss and Heisenberg, 1993; Martin et al., 1999; Strauss, 2002; Poeck et al., 2008; Triphan et al., 2010), cockroaches (Guo and Ritzmann, 2013; Guo et al., 2014; Martin et al., 2015) and crickets (Kai and Okada, 2013) and is considered as site for action selection and goal-directed behavior (Libersat and Gal, 2013; Strausfeld and Hirth, 2013; Barron et al., 2015; Fiore et al., 2015; Barron and Klein, 2016). A role of the CX in visual learning of patterns and spatial features has been shown in various behavioral assays using fruit flies (Liu et al., 2006; Neuser et al., 2008; Wang et al., 2008; Pan et al., 2009; Hou et al., 2011; Ofstad et al., 2011; Kuntz et al., 2012, 2017).

In this study, we used the transient and local anesthetic procaine to selectively silence neural activity in these three brain regions. Procaine is a reversible blocker of voltage-gated Na^+^- and other voltage-gated channels to a lesser degree and has been established as a means to study olfactory learning and memory in honey bees (Muller et al., 2003; Devaud et al., 2007, 2015). Procaine has also been utilized to show that silencing the central body reduces spontaneous walking and optomotor responses (Kathman et al., 2014; Kaiser and Libersat, 2015). Our expectation was that mushroom bodies are needed for this form of visual conditioning with a strong operant component. This allowed the bee to learn from consequences of her behavior and not only from a stimulus-stimulus pairing. Interrupting processing in the collar region and blocking the further processing in the output regions of the mushroom bodies could lead to an impairment in performance in aversive visual learning which can be measured in the APIS assay. We hypothesized further that learning of the stimulus-shock pairing would remain intact when the central complex was anesthetized but the reaction of running away from the stimulus would be impaired. We discuss how our results will contribute to uncovering mechanisms underlying visual learning in insects.

## Materials and methods

### Animals and surgical procedure

For all experiments, honey bees were collected from two established queen-right colonies at Macquarie University in Sydney, Australia. Foragers were collected at the hive entrance while leaving for a foraging bout. Bees were immobilized on ice and harnessed in PER tubes (Bitterman et al., 1983; Felsenberg et al., 2011). To prepare the animals for injections, the bee's neck was filled with soft dental wax to prevent movement of the head. A stripe of wax was positioned loosely over the antennae to prevent their movement during the operation.

For MBC injections, we entered through the ocellar tract. The lens of the median ocellus was carefully pushed outwards with the tip of a micro-scalpel and a small incision was made into the neurilemma sheath covering the brain to ease entering of the micropipette.

To access the brain for intracerebral injections (VL and CX), a window was cut into the head capsule with three cuts: One above the antennal stems (dorsal), one below the median ocellus (ventral), and one at the border of the right eye (Devaud et al., 2007). The created flap was opened and held in place with soft dental wax. The glands and trachea above the brain were carefully moved aside and a small incision was made into the neurilemma above the target structure to enable a smooth entry of the micropipette during injections. After injections the flap was carefully released to close the window and sealed with a drop of eicosane (Sigma-Aldrich Australia) melted at ~35°C). For detailed demonstration of the procedure please refer to Søvik et al. (2016).

### Injections

In the following study four different treatment groups were compared: procaine-injected animals (procaine/proc), saline-injected animals (vehicle/veh), animals that underwent the operation and injection procedure without having any solution injected into the brain (sham), and non-treated animals (NT), which were directly transferred to the chamber after catching.

To locally and temporarily inhibit neural activity, the drug procaine was used. In the honey bee procaine reduces Na^+^- and K^+^-currents and spiking activity in mushroom body neurons (Devaud et al., 2007). Procaine HCl (Sigma-Aldrich Australia) was dissolved in physiological saline (7.54 g/L NaCl, 0.448 g/L KCl, 0.872 g/L MgCl_2_ × 6 H_2_0, 0.735 g/L CaCl_2_ × 2H_2_0, 54.72 g/L Sucrose, 4.95 g/L D-glucose, and 2.38 g/L HEPES, pH = 6.7, 500 mOsm, Sigma-Aldrich Australia, see Burger et al., 2013) as a stock solution of 40% (w/v). On the day of the experiment, the solution was diluted with additional saline to create a 20% (w/v) procaine solution. Physiological saline was also used as a control solution. To identify the injection site afterwards, both solutions contained 0.5 mg/ml dextran Alexa fluor 546 or dextran Alexa fluor 568 (10.000 MW, Molecular probes by Life technologies, Carlsbad, CA, USA). Microinjections were performed with a microinjector (Eppendorf, Hamburg, Germany) and an electronic micromanipulator (Luigs & Neumann Feinmechanik und Elektrotechnik, Ratingen, Germany). Micropipettes were pulled from glass capillaries (World Precisions Instruments, Sarasota, FL, USA) using an electrode puller (Scientific & Research Instruments, Karnataka, India). The tips were broken to an outer diameter of 10–15 μm. The injection volume was adjusted and rechecked both before and after every animal by measuring a droplet injected into mineral oil.

**Figure 1:**
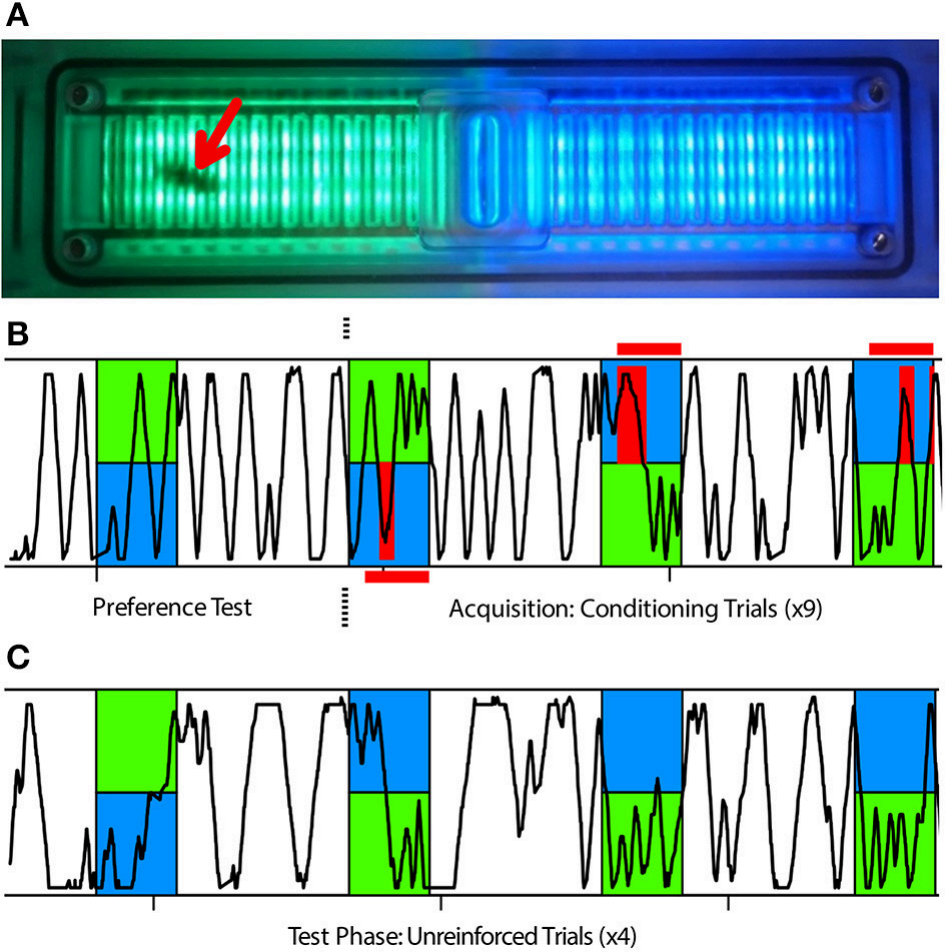
**APIS learning assay used in this study. (A)** The APIS chamber can be illuminated with two different light fields of varying wavelengths and intensities; in this case light appearing green to humans and light appearing blue to humans. The chamber is equipped with an electrifiable grid to deliver 10 V shocks to the bee's feet and with infrared sensors to automatically track the bee's movement. A bee in the chamber (red arrow) could only move in a straight line, either toward or away from a stimulus, and turns were scored as a reversal of direction as detected by the infrared sensors. **(B,C)** Typical running trace of a bee in the chamber. Blue and green indicate illumination wavelength and red indicates when shocks were available (red horizontal bars) or delivered (red vertical bars) to the bee. Blue light was always illuminating the half of the chamber in which the bee was located at light-onset. **(B)** After an acclimatization period of 15 min post-injection, the bee was exposed to 14 s of both green and blue illumination as a preference test. The bee was then subjected to nine conditioning trials in which, after 3 s of illumination, the bee experienced shocks on the blue side for another 11 s, but not on the green side. **(C)** Subsequently, the bee was tested four times with 14 s of illumination without shocks to determine the post-training response to blue and green light fields.

Injections into the MBC occurred via the ocellar tract of the median ocellus. The micropipette was brought to the opening of the removed lens and then finely adjusted until the micropipette was just above the incision made earlier. The micropipette was then inserted to a maximum injection depth of ~215 μm and a volume of ~2 nL was injected. The micropipette was removed and the bee was quickly transferred into the conditioning chamber (Figure 1A).

To target the center of the VL, ~1 nL of solution was injected into each lobe at a depth of ~60 μm and at an angle of 68–70° relative to the brain surface. A stereomicroscope fluorescent adapter was then used to visualize the injection site (Green- Light and Filter Set; NIGHTSEA, Lexington, MA, USA). Successful injections were identified by spreading of the fluorescent dye throughout the VL. To target the CX, ~0.5 nL of solution was injected at a depth of ~330 μm and at an angle of 68–75° relative to the brain surface; entering at the midline between the VLs. Successful injections were identified using laser scanning confocal microscopy (see below).

### Behavioral assay

Honey bees were conditioned in the APIS chamber, designed and manufactured at the University of Konstanz, Germany with an aversive visual conditioning paradigm established in (Kirkerud et al., 2017). Tracking of the bee and delivery of stimuli in APIS are fully automated which eliminates human error or bias. Due to the design of the chamber, bees can only move in almost straight lines, either toward or away from a stimulus, and any turn made by the animal is tracked as a complete reversal by the sensors. Shock and light stimuli were controlled with a script loaded into the system software. The program utilizes sensor feedback to determine the bee's location and initiates stimuli at specified time points. The operation of the chamber and the assay used are similar to methods used earlier in flies (Zars et al., 2000; Claridge-Chang et al., 2009).

Following injection, the bee was quickly placed into the chamber and allowed to acclimate for 15 min while freely moving around in the dark. The conditioning protocol consisted of one unreinforced preference test followed by nine reinforced training trials (Figure 1B), and ending with four unreinforced test trials (Figure 1C). In each trial, a blue light field (λ^B^ = 465 nm, Luminous intensity: 105 mcd) was switched on in the half of the chamber where the bee was situated and a green light field (λ^G^ = 525 nm, Luminous intensity: 119 mcd) illuminated the opposite half. All trials lasted 14 s and were presented at regular intervals of 44 s (from onset to onset). For the training trials, electric shock pulses (10 V, 4 Hz, 100 ms) were activated 3 s after light onset. These shock pulses were delivered to the feet of the bee through the metal grid as long as movement sensors on the blue side were triggered. This meant that the bee could either escape the shocks by crossing from the shock-predicting blue side to the safe green side or potentially avoid them completely by escaping within 3 s and remain on the green side until the end of the trial. Since bees were always located on the blue half at trial onset (Figure S3), there was an inherent bias in the calculated preference toward this side. Once the behavioral assay was complete, the bee was quickly placed onto ice and anesthetized for dissection.

### Histology and imaging

Once anesthetized, the bee's head capsule was opened and the brain was removed in 0.1 M PBS (Sigma-Aldrich Australia) using forceps and a fresh breaker-blade piece. Whole brains were fixed in 4% paraformaldehyde (Electron Microscopy Sciences, Hattfield, PA, USA) in 0.1 M PBS overnight in a chilled room (16°C). Brains were then washed in 0.1 M PBS (3 × 10 min) at room temperature (22°C) and stored in the fridge (4°C). Samples were either washed daily with fresh 0.1 M PBS or they were processed immediately for histology.

Whole brains were incubated in 250 μL DAPI (2 μg/ml, Sigma-Aldrich Australia) in 0.1 M PBS and 0.2% Triton-X 100 (Sigma-Aldrich Australia) overnight. Brains were then washed in 0.1 M PBS (3 × 10 min) followed by an ethanol dehydration series (i.e., 50, 70, 90, 98, 100, 100% 10–30 min each step) and cleared in methylsalicylate (Sigma-Aldrich Australia).

Brains were then mounted on previously prepared slides with a cavity well. Wells were created with glass cover slips (Marienfeld-Superior, Lauda-Koeningshofen, Germany) and custom made aluminum slides (manufactured at the University of Konstanz, Germany) secured together using DPX mounting medium (Sigma-Aldrich Australia). Cleared brains were mounted in the well using DPX mounting medium and sealed with another cover slip.

**Figure 2:**
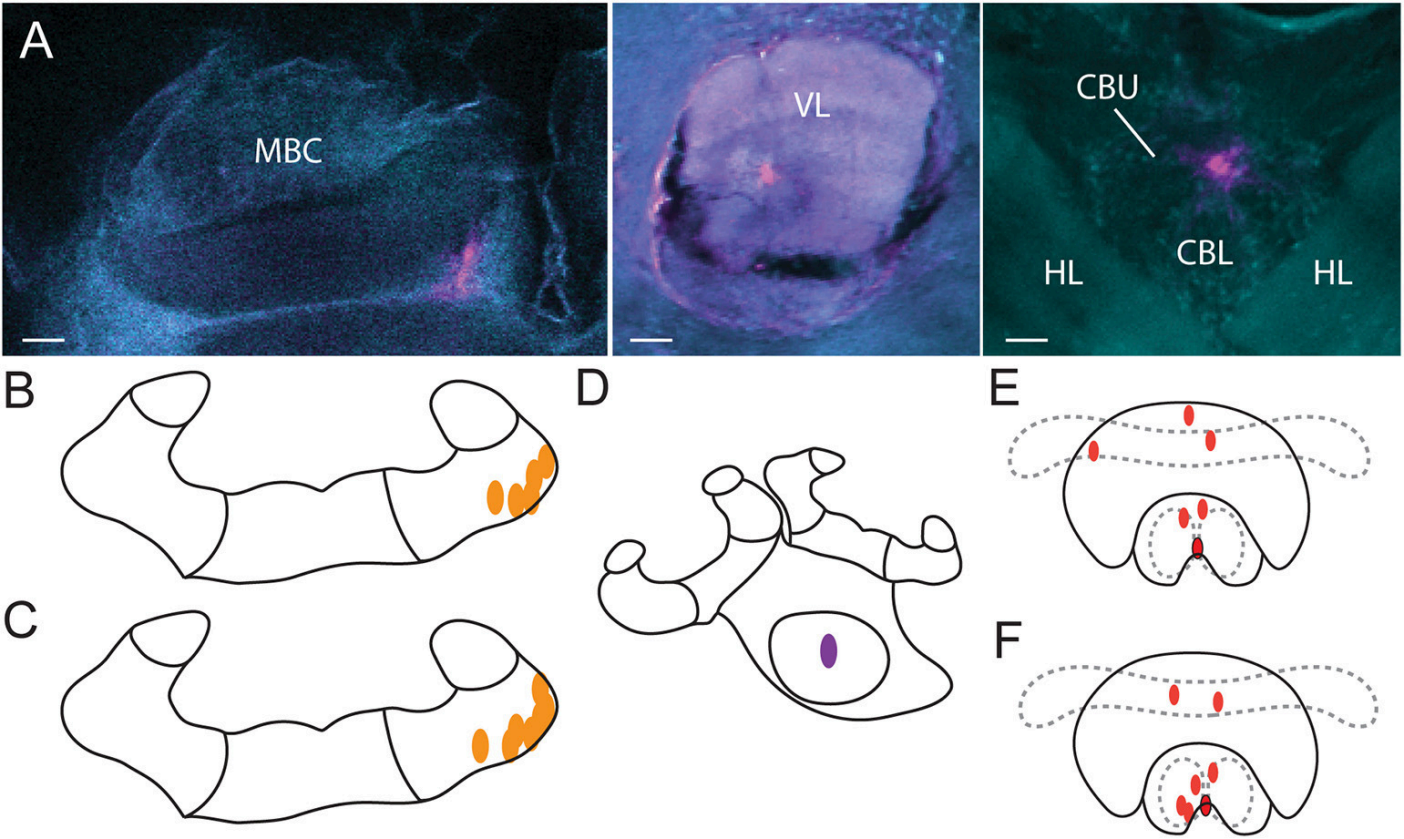
**Injection sites. (A)** Alexa dye injections are shown in magenta (false color) in the MBC (left), VL (middle) and the CX (right). A DAPI-counterstain and auto-fluorescence of the brain tissue (false colored in cyan) allowed us to identify brain neuropils. Orientation of all three scans was aligned with rostral (neuraxis) facing upwards. Injections of vehicle **(B)** and of procaine solution **(C)** into the MBC as identified by the CLSM scans. Injections into the VL **(D)** were identified visually with fluorescent light and were all located in the center. Injections of vehicle **(E)** and of procaine solution **(F)** into the central body (red dots) and injections located at the border of the lower division of the central body with spread into the noduli (red dots with black border). MBC, mushroom body calyces; VL, ventral lobes; HL, horizontal lobes; CBU, upper division of the central body; CBL lower division of the central body; Scale bar = 30 μm.

Samples were imaged (4.77 μm slice) using an Olympus Fluoview inverted confocal microscope (FV-1000 IX81) located at Macquarie University in Sydney, Australia. DAPI staining and auto-fluorescence of the tissue was used to identify the neuropils and determine the location of the injection site marked by the Alexa dye (Figure 2).

All injections in the CX group were located in the central body (Figures 2E,F). One injection in the vehicle group (Figure 2E, red dot with black border), and one injection in the procaine group (Figure 2F, red dot with black border), was located at the border of the lower division of the central body and some dye was also found in the noduli; indicating that those areas were possibly affected as well. Since the performance in APIS was very similar for both injection sites, results were presented for all combined CX injections.

### Data analysis

The data was analyzed and graphed using R 3.3.2 (R Core Team, Vienna, Austria) and RStudio 1.0.136 (RStudio Inc., Boston, MA, USA) with a custom written script. As a measurement for learning, the Performance Index (PI) was calculated: difference between time spent on the green side of the chamber and time spent on the blue (shocked) side of the chamber divided by the total trial time:

$$\begin{array}{l} PI=\frac{\;t\left(green\right)-t\;\left(blue\right)}{t\left(green\right)+t\;\left(blue\right)} \end{array}$$

**Figure 3:**
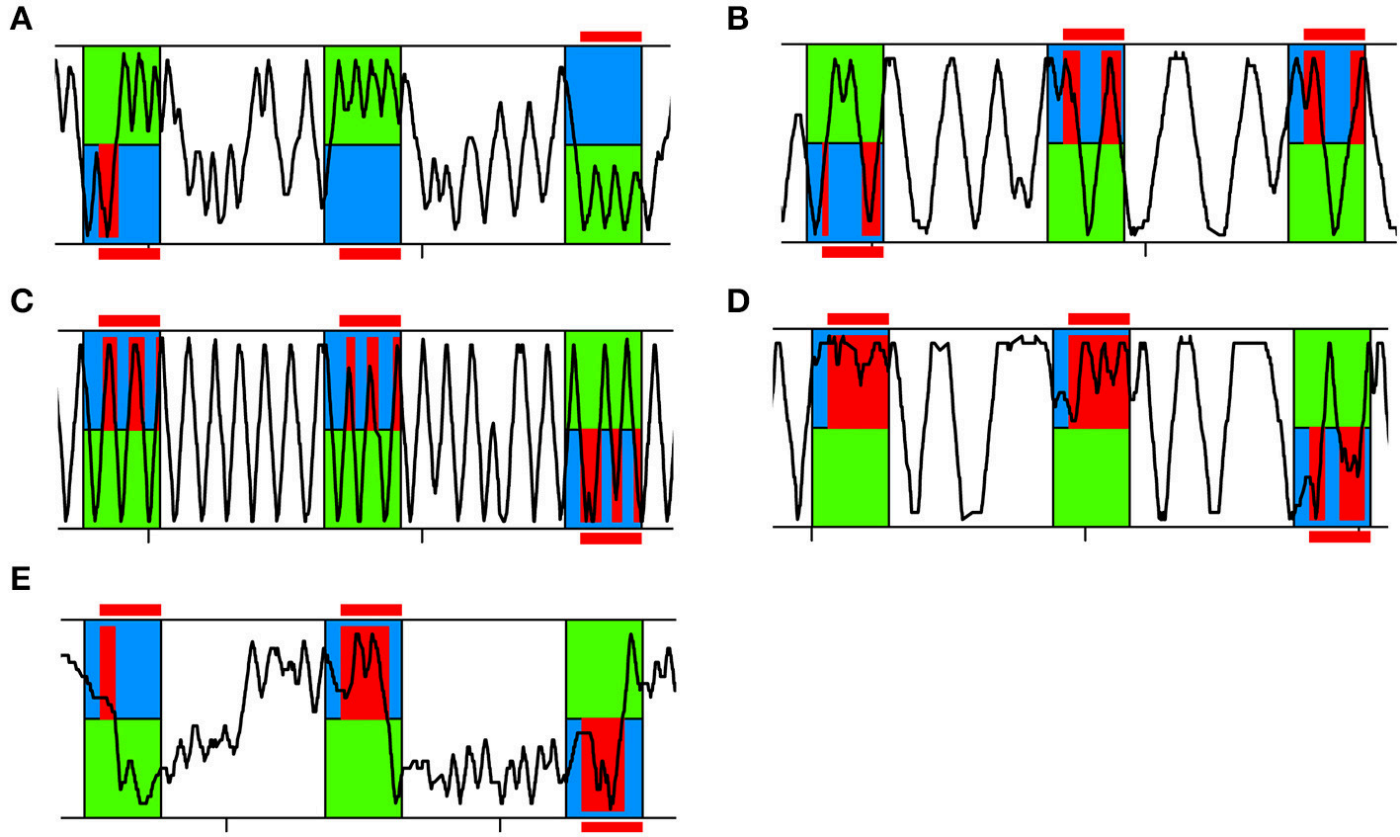
**Representative running traces of individual bees in APIS**. Three training trials are shown. The bee was exposed to 14 s of blue and green light fields. After a 3 s delay the bee experienced shock when located on the blue side (red). **(A)** Typical running trace of a bee spending more time on the green side than on the blue side, thus achieving high Performance Indices (PIs). **(B)** Typical running trace of a bee spending more time on the blue side than on the green side, thus achieving low PIs. **(C)** Typical running trace of a bee with an equal number of reversals on the green and blue side, thus achieving a Reversing Difference close to zero. **(D)** Representative running trace of a bee reversing more often on the blue side than on the green side, thus achieving a negative Reversing Difference. **(E)** Typical running trace of a slowly responding bee taking a long time to cross over to the green side at the beginning of each trial and after light-onset, thus achieving a high Crossing Latency.

This resulted in a variable ranging from −1 to 1, where positive values indicate that the bee spent more time on the safe side than on the shocked side, negative values the opposite. A bee that had learnt to associate the blue light with shock would run away from the blue side shortly after light-onset and avoid returning to the blue side. As a consequence, the relative time spent on the green side increased leading to higher PI-values (Figure 3A). A bee that had not learnt, spent equal amounts of time on each side or more time on the blue side. A bee that had not learnt, would be expected to have lower PI-values (Figure 3B).

To investigate the movement pattern of the bee in more detail we further analyzed how many reversals of direction were performed in the chamber. We analyzed the total number of reversals per trial and the Reversing Difference: number of reversals performed on the blue side subtracted from the number of reversals performed on the green side of the chamber divided by the total number of reversals:

$$\begin{array}{l} Reversing\;Difference=\frac{\;reversals\left(green\right)-reversals\;\left(blue\right)}{reversals\left(green\right)+reversals\;\left(blue\right)} \end{array}$$

A bee that had learnt to avoid returning to the blue side typically ran back and forth on the green side (Figure 3A). If a bee had not learnt to avoid the blue side, we found two patterns: either she was running back and forth in the whole chamber (Figure 3C) or she was running back and forth on the blue side (Figure 3D). In the former case, the number of reversals performed would be equal for both sides (Reversing Difference close to zero). In the latter case, the number of reversals performed was higher on the blue side than on the green side (negative Reversing Difference).

As another parameter for learning performance as well as to evaluate the reaction to the shock-paired light, we analyzed how fast an animal would cross over to the green side after light-onset (Crossing Latency). If the bee managed to cross over under 3 s, she could completely avoid being shocked due to the delay of the shock-onset after light-onset, assuming she would not then return to the blue side (Figure 3A, second and third trial shown). If Crossing Latency was higher than 3 s she would experience shocks on the blue side (Figure 3E).

For statistical analysis of PI, Speed, Reversing Difference, Crossing Latency and Position in Chamber (at light-onset), the calculated data were fitted to linear mixed models with trial and treatment (procaine, vehicle, sham, NT) as fixed effects and bee identity as a random effect to correct for repeated measurements in the training, as well as the test phase (lme function in the R nlme package, Pinheiro et al., 2016). For statistical analysis of Reverses per Trial the calculated data were fitted to generalized linear mixed models (Poisson distribution) with trial and treatment (procaine, vehicle, sham, NT) as fixed effects and bee identity as a random effect to correct for repeated measurements in the training, as well as the test phase (glmer function in the R lme4 package, Bates et al., 2015). Statistical differences were determined *post-hoc* with the Tukey's range test using the R multcomp package (Hothorn et al., 2008). Since bees with lower speeds could not perform well in this assay in which performance is based on movement, animals with lower speeds than 2.1 cm/s were excluded from the analysis (Figure S1).

## Results

### Control animals learned to remain on the green side

**Figure 4:**
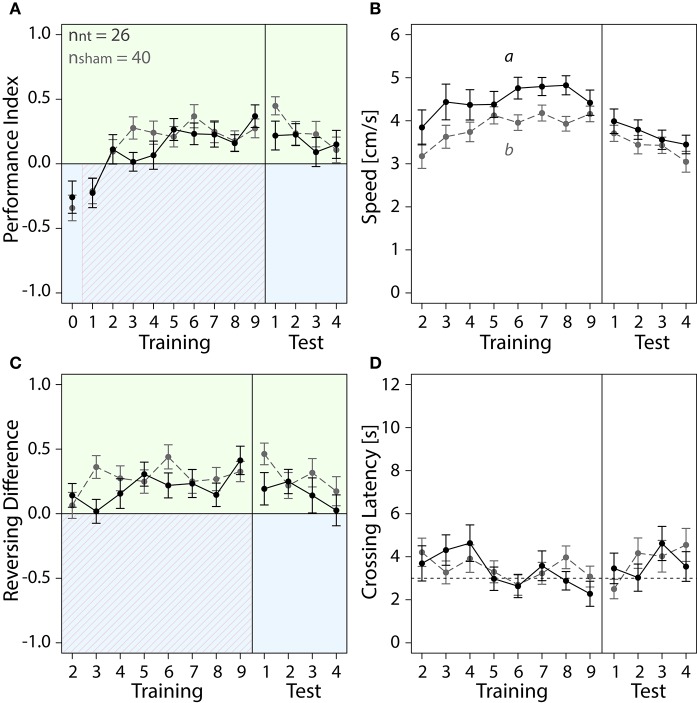
**With training, bees of sham and NT control groups learned to spend more time on the safe green side than the shocked blue side**. Means ± SEM are plotted for all variables. Non-treated animals (NT) are shown in black, sham-treated animals (sham) in gray. No effect of the different injection methods used for the different regions on any of the four variables shown was found (ANOVA, *p* > 0.05). Sham-treated animals were therefore pooled into one group to compare with NT animals. Significant treatment effects determined with an LMM (*p* < 0.05, Table S1) are indicated with letters a and b. Bees were subjected to one preference test (0) nine training trials and four test trials. Control animals spent more time on the green side and avoided the shock-paired blue side (shocked period indicated by red diagonal lines) after a few trials. **(A)** No effect of treatment on Performance Index was found in training or in the test phase (Table S1). **(B)** An LMM indicated a significant effect of treatment on speed (Table S1). After one conditioning trial, speed was lower in sham-treated animals than in NT-animals in the training (*post-hoc* Tukey HSD, *z* = −2.188, *p* = 0.03), but no significant effect of treatment on speed was found in the test phase (Table S1) **(C)** Number of reversals on the green side was higher after one conditioning trial. No significant effect of treatment was found in training or in the test phase (Table S1). **(D)** Crossing Latency approached the 3-s threshold (horizontal dashed line) over the course of training, which corresponds to the delay between light-onset and shock-onset. **(A)** No significant effect of treatment on Crossing Latency was found for training or in the test phase (Table S1).

In this study, we investigated color learning and how the animal's behavior in response to a learned stimulus changed. We first studied the behavior of the non-treated (NT) and sham-treated control groups. NT and sham-treated bees both developed a preference for the safe green side after few trials of color-shock conditioning (Figure 4). For both control groups PIs increased over the course of training (Figure 4A). PIs corresponded to around 39% of the first trial spent on the green side which increased to 61% (NT) and 72% (sham) in last trial. Increase of PIs from the first to the last trial was significant for both, NT animals (paired *t*-test, df = 25, *t* = −2.682, *p* = 0.013) and for sham-treated animals (paired *t*-test, df = 39, *t* = −5.4861, *p* < 0.001). In the test phase both groups continued to spend more time on the green side (Figure 4A).

We further explored how running and reversing in the chamber changed in response to the first light-shock pairing. Sham-treated animals were slower than NT-animals in the training but not in the test phase (Figure 4B). After five conditioning trials both groups performed on average three to five more reversals on the green side (Figure 4C). The total number of reversals performed in the chamber remained constant in that period (Figure S2A). Both groups crossed over to the green side after 2 to 4 s into the trial (Figure 4D). In the last training trial 20 out of 26 NT-animals and 21 out of 40 sham-treated animals crossed over under 3 s (data not shown). Taken together, after learning to associate blue light with shock the control bees ran away from the blue side before or shortly after shock-onset and thereafter ran back and forth on the green side.

### Procaine injections into the MBC did not impair performance in the visual learning paradigm

**Figure 5:**
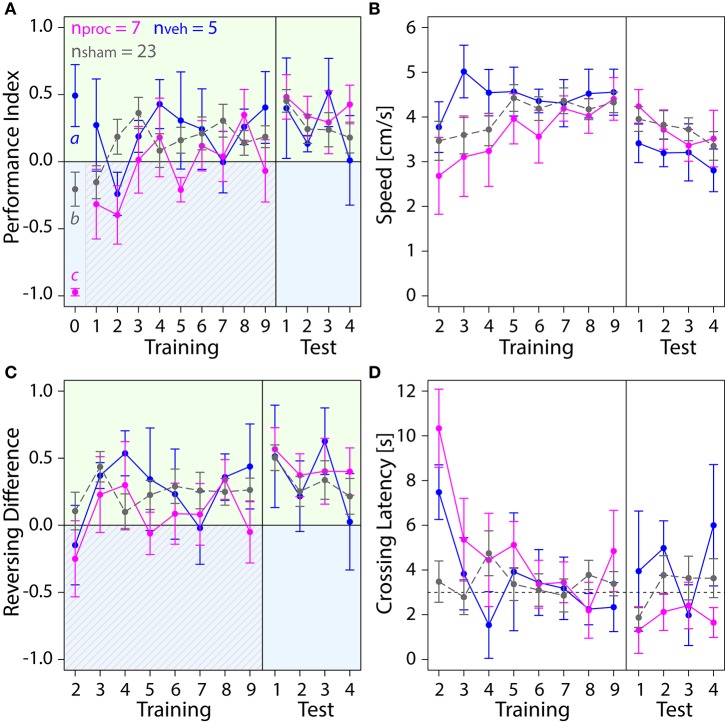
**Comparison of behavior in the APIS assay for bees injected with the vehicle (blue) or procaine solution (magenta) into the MBC, or sham-treated bees (gray)**. All groups learned to spend more time on the green side. Means ± SEM are plotted for all variables. Significant treatment effects determined with an LMM (*p* < 0.05, Table S1) are indicated with letters a, b, and c. Bees were subjected to one preference test (0) nine training trials and four test trials. **(A)** An LMM indicated an effect of treatment on Performance Index (PI) in the preference test (Table S1). Treatment comparison with a Tukey HSD *post-hoc* test revealed differences in PIs of vehicle and sham groups (*z* = 2.631, *p* = 0.02), PIs of procaine and sham groups (*z* = −3.310, *p* = 0.003) and PIs of procaine and vehicle groups (*z* = −4.657, *p* < 0.001). An LMM indicated a significant difference between PIs of procaine and sham groups in training (Table S1), but a Tukey *post-hoc* test, which corrects for multiple testing indicated no difference between PIs of these groups (*z* = 2.080, *p* = 0.09). No effect of treatment on PIs was found for the test phase (LMM, Table S1). All bees spent more time on the green side and avoided the shock-paired blue side (shocks indicated by diagonal lines) after a few trials. **(B)** Speed did not differ between experimental groups (LMM, Table S1). **(C)** Number of reversals on the green side was higher after one conditioning trial. No effect of treatment on Reversing Differences was found in training or in the test phase (Table S1). **(D)** Crossing Latency approached the 3-s threshold (horizontal dashed line) over the course of training, which corresponds to the delay between light-onset and shock-onset. No significant effect of treatment on Crossing Latency was found for training or in the test phase (Table S1).

We then examined how silencing of neurons of a collar region in the MBC with procaine injections changed the bees' behavior in the APIS assay (Figure 5). Procaine- and vehicle-injected animals were compared to sham-treated animals which were operated on in the same way. Overall, we observed no impairment of the bees' performance in the learning assay due to the injections. All bees were able to avoid the blue side after a few trials and moved normally. Curiously, we found a difference between PIs for all three groups in the preference test (Figure 5A). However, this did not seem to have an effect on the training where all groups performed similarly. Neither speed (Figure 5B), Reversing Differences (Figure 5C), Reversals per Trial (Figure S2B) or Crossing Latencies (Figure 5D) after the second trial were affected by injections (Table S1).

### Procaine and vehicle injections into the VLs impaired performance in the visual learning assay

**Figure 6:**
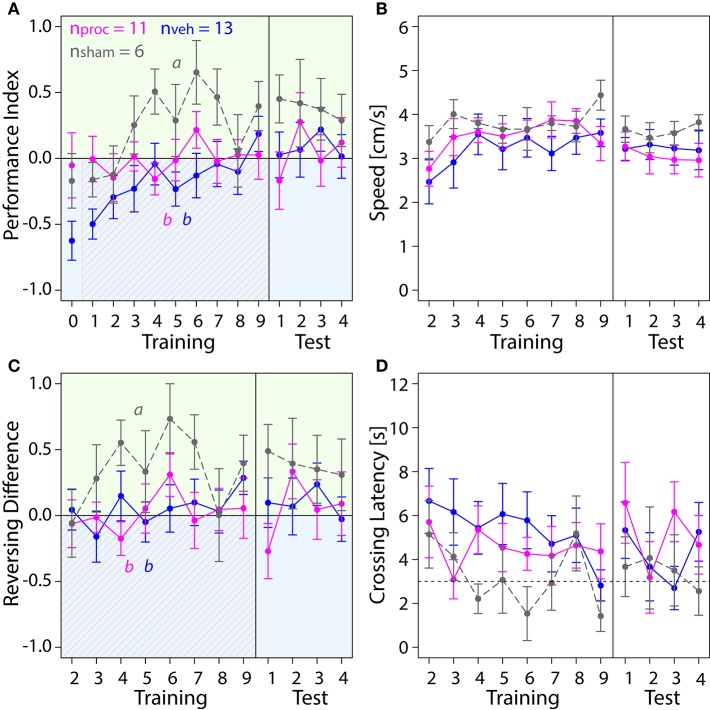
**Comparison of behavior in APIS for bees injected with vehicle (blue) or procaine solution (magenta) into the VLs, or sham-treated bees (gray)**. Learning to differentiate the shock-paired blue side and the safe green side was impaired in procaine and vehicle groups. Means ± SEM are plotted for all variables. Significant treatment effects determined with an LMM (*p* < 0.05) are indicated with letters a and b. Bees were subjected to one preference test (0) nine training trials and four test trials. **(A)** An LMM indicated an effect of treatment on Performance Index (PI) in the training but not in the test phase (Table S1). Treatment comparison with a Tukey HSD *post-hoc* test showed differences in PIs of vehicle and sham groups (*z* = −4.217, *p* < 0.001) and PIs of procaine and sham groups (*z* = −2.638, *p* = 0.02). **(B)** Speed did not differ between experimental groups (LMM, Table S1). **(C)** Reversing Differences were affected by treatment in the training but not in the test phase (LMM, Table S1). Treatment comparison with a Tukey HSD *post-hoc* test showed differences in Reversing Difference of vehicle and sham groups (*z* = −3.107, *p* = 0.005) and Revering Differences of procaine and sham groups (*z* = −3.567, *p* = 0.001). **(D)** Crossing Latency approached the 3-s threshold (horizontal dashed line) over the course of training, which corresponds to the delay between light-onset and shock-onset. No significant effect of treatment on Crossing Latency was found for training or in the test phase (Table S1).

Next, we investigated which role the VL as part of the MB output played in visual learning (Figure 6). Surprisingly, injections into the VL with either, procaine or vehicle solution resulted in impairment of color learning. Both groups achieved mean PI-values around zero, indicating that they spent equal amount of time on both sides (Figure 6A). This was not the case in sham-treated animals, which preferred the safe green side after two trials. Thus injection of the vehicle (with or without procaine), but not the insertion of the micropipette itself impaired learning of the light-shock pairing. Lower PIs in vehicle and procaine groups were not the result of impaired locomotion, since speed (Figure 6B) was not affected by treatment (Table S1). Furthermore, vehicle and procaine groups with injections into the VLs showed equal number of turns on the green side as on the blue side (Figure 6C), while Reversals per Trial (Figure S2C) remained unaffected. This indicated that the bees were either running back and forth from one side of the chamber to the other or were spending equal amounts of time running back and forth on each side. However, Crossing Latencies (Figure 6D) were found not to be significantly different (Table S1). Thus, vehicle- and procaine-treated bees ran away from the shocks after a similar delay as sham-treated bees in most trials.

### Procaine injections into the CX changed behavioral responses in the visual learning paradigm

**Figure 7:**
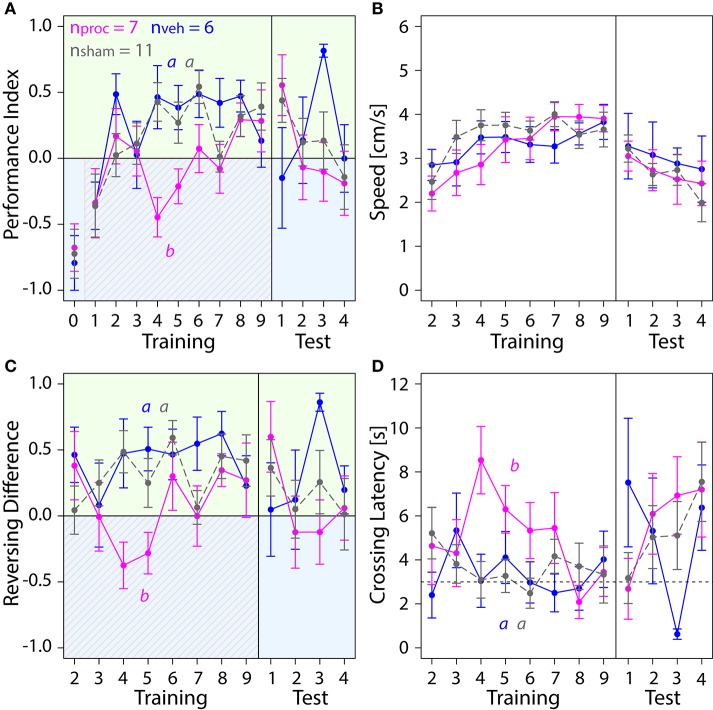
**Comparison of behavior in APIS for bees injected with vehicle (blue) or procaine solution (magenta) into the CX, or sham-treated bees (gray)**. Bees injected with procaine into the CX did not run away from the shock-paired blue side. Means ± SEM are plotted for all variables. Significant treatment effects determined with an LMM (*p* < 0.05, Table S1) are indicated with letters a and b. Bees were subjected to one preference test (0) nine training trials and four test trials. **(A)** Performance Indices (PIs) were affected by treatment in the training but not in the test phase (LMM, Table S1). Treatment comparison with a Tukey HSD *post-hoc* test showed differences in PIs of procaine and sham groups (*z* = −2.512, *p* = 0.03) and PIs of procaine and vehicle groups (*z* = −3.052, *p* = 0.006). **(B)** Speed did not differ between experimental groups (LMM, Table S1). **(C)** An LMM indicated an effect of treatment on Reversing Differences in the training but not in the test phase (Table S1). Treatment comparison with a Tukey HSD *post-hoc* test revealed differences in Reversing Difference of procaine and sham groups (*z* = −2.629, *p* = 0.02) and Reversing Differences of procaine and vehicle groups (*z* = −2.995, *p* = 0.008). **(D)** In vehicle and sham groups Crossing Latency approached the 3-s threshold (horizontal dashed line) over the course of training, which corresponds to the delay between light-onset and shock-onset. An LMM revealed an effect of treatment on Crossing Latency in the training but not in the test phase (Table S1). Treatment comparison with a Tukey HSD *post-hoc* test showed differences in Crossing Latencies of procaine and sham groups (*z* = 2.467, *p* = 0.04) and Crossing Latencies of procaine and vehicle groups (*z* = 2.532, *p* = 0.03).

Lastly, we explored how an animal's performance in the APIS-chamber was changed by silencing neural activity in the CX with procaine (Figure 7). Procaine-treated animals did not show a preference for the green side in the middle trials of the training. Rather, they remained on the shock-paired blue side longer than vehicle- and sham-treated animals. PIs were lower in procaine-treated animals in the training (Figure 7A). In fact, these bees spent 60–70% of the trial duration on the blue side in the middle of the training. Hence, the animals either did not leave the blue side or returned to the blue side more often. This behavior was not due to an impairment in locomotion since we found no differences in speed (Figure 7B) in the training (Table S1). However, toward the end of the training and in the test phase procaine-treated bees preferred the green side and PIs were similar to those found for vehicle- or sham-treated bees. We further explored if the ability to reverse in the chamber might have been affected. Procaine-treated bees did not reverse in the chamber less often than vehicle- or sham-treated bees (Figure S2D) (Table S1). But they performed on average three to four more reversals on the blue side than on the green side in the middle trials of training (Figure 7C). In contrast, vehicle- and sham-treated bees performed on average three to five more reversals on the green side in the same trials. Additionally, Crossing Latency was found to be on average 6 to 8 s in the middle trials for procaine-treated bees (Figure 7D). This was about twice as long as Crossing Latencies found for vehicle-treated and sham-treated bees and around 40–60% of the trial duration. Thus, procaine-treated bees did not leave the blue side even when the shocks were delivered for more than 3 s. Differences in Crossing Latencies were not due to different starting positions at light-onset in the training (Figure S3D) (Table S1).

## Discussion

About a decade ago the MBs were believed to process mainly olfactory information to generate meaningful associations to other stimuli. The CX was believed to primarily process visual and spatial information. Amongst other recent studies this study has shown this division might not necessarily be so clear. Our data indicate that the VLs as part of the MB output as well as the CX are involved in differential visual learning in the APIS assay.

### Mushroom body function was required for visual learning with a choice component

Control bees escaped the shock-paired light field and avoided returning to it after only a few conditioning trials (Figure 4). These results were congruent with data obtained from untreated forager bees conditioned in the same assay in Konstanz, Germany (Kirkerud et al., 2017), and confirms the robustness of the paradigm across continents. While the operation and injection is an invasive procedure, we found that sham-treated animals recovered well and showed no deficits in learning performance compared to NT animals. In contrast, bees with silenced VLs escaped the shock-paired light field but failed to remain in the safe light field (Figure 6). Instead, they ran back and forth in the chamber resulting in lower PIs. This behavior indicated that they most likely failed to associate one light field with danger and the other light field with safety. We found a similar behavior in bees injected with the vehicle only. A similar phenomenon was found when injections of PBS into the MB lobes led to a reduced performance in olfactory reversal learning in comparison to injections into the calyces (Boitard et al., 2015). However, no effect of the vehicle was found when observing neural activity changes due to injections using calcium imaging (Girardin et al., 2013).

When targeting one collar region of the MBC with procaine we found no deficits in performance (Figure 5). But since the honey bee collar region receives color input (Ehmer and Gronenberg, 2002; Gronenberg and Lopez-Riquelme, 2004) and the VLs were clearly involved in visual learning in APIS, it is possible that silencing neurons in only one of the eight collar regions in all MBCs might not have been sufficient to impair performance in the APIS assay. Further studies impacting all collar regions are necessary to clarify, but technically this would be extremely tricky to do.

In freely moving fruit flies, MB function was required for a visual paradigm with color stimuli and aversive reinforcement (Vogt et al., 2014, 2016). Similar to the paradigm presented here, blue and green light fields were presented simultaneously rather than sequentially. These findings stand in contrast to other studies implicating no involvement of the MBs in visual learning. Mutant flies (*Drosophila melanogaster*) with severely underdeveloped MBs and interrupted MB input were either conditioned by being shaken while illuminated with one color (Heisenberg et al., 1985) or trained with heat stimuli in a differential visual assay while being tethered in a flight simulator (Wolf et al., 1998). In both cases, mutant flies showed no learning deficits. In the latter case the fly was able to terminate the heat stimulus by turning left or right until the adjacent 90°-quadrant of the arena was faced and the arena was then illuminated with light of a different color. This suggests that the MBs are involved in color learning which includes a choice situation rather than learning of sequentially presented color stimuli in a differential paradigm. Indeed, it has been shown that MBs are required to make a choice of responding to conflicting information of color and shape or color and position based on saliency (Tang and Guo, 2001; Zhang et al., 2007).

In both, bees and flies the dominant input to the MBs is olfactory, but it appears that MBs are also crucial for learning of visual information in bees in a binary-choice assay. Strausfeld (2012) and Farris (2015) argue that processing of visual information in the MB in insects is largely driven by the ecological relevance in the animal's life and the nature of visual input received. Large MBs with developed calyces are therefore not limited to species which rely predominately on olfactory information to navigate in their environment. They can also be found in aquatic beetle species which navigate mainly by vision (Lin and Strausfeld, 2012). It remains to be investigated if the MBs play a role in visual learning in other insect orders as well.

### Silencing neurons in the central complex affected the behavioral response

We also found that silencing of neurons in the CX led to a change in behavior (Figure 7). Procaine-treated bees spent more time in the safe light field than on the shock-paired light field in the second and third trials and in the end of the training. This indicates, that learning of the light-shock pairing might still have been present. In the middle of the training period, however, procaine-treated bees remained on the shock-paired side of the chamber even after several seconds of shocks being delivered. This was not a result of an impaired ability to initiate reversals or an inability to walk in a straight line (Figure S2D). Nor was it caused by a major deficit in locomotion since speed was not found to be affected by procaine-injections, and rather bees appeared unable to execute an avoidance of the shocked light field.

But why was the effect not visible in the first learning trials? It seems very unlikely that procaine was only active in the middle trials of the training. Cockroaches with central bodies silenced by procaine showed deficits in locomotion and optomotor responses immediately after injections (Kathman et al., 2014; Kaiser and Libersat, 2015). Another explanation is that the response in the first trials might have mainly been driven by a direct reaction to the shocks, resulting in a short-lasting reflex-like escape maneuver. Initial responses to the shock could have been initiated by more direct and faster-processing “escape-pathways” generating a quick behavioral response to an obnoxious stimulus without involving the CX. Various escape reactions in insects have been proposed that bypass the higher processing centers of the brain (Horridge, 1962; Card, 2012). Is it possible that silencing of the CX only interfered with coordinating a motor response to a learned visual stimulus, but not an escape response from an aversive stimulus? In this case, a learned response to the blue light field would have been impaired but not the response to the shock itself. Toward the end of the training the procaine-effect seemed to have worn off, since the bees rapidly increased the proportion of time spent on the safe green side.

The CX has been implicated as the site to generate goal-directed behavior and to modulate movement in insects (Strausfeld and Hirth, 2013; Barron et al., 2015; Plath and Barron, 2015). Various studies have shown that the CX is crucial for spatial orientation memory (Neuser et al., 2008; Kuntz et al., 2012, 2017), visual pattern memory (Liu et al., 2006; Hou et al., 2011) and visual place learning (Ofstad et al., 2011) in fruit flies. A recent study has shown that a group of neurons in the ellipsoid body (part of the CX in the fruit fly) represents the orientation of the animals in relation to a visual stimulus (Seelig and Jayaraman, 2015). Taken together, the CX clearly has a role in visual learning and memory involving spatial orientation of the cues in fruit flies and possibly in other insects. We propose that the CX might also initiate the appropriate responses to learned stimuli which are processed by the MBs such as color stimuli.

### Information about a learned stimulus might be conveyed indirectly to the central complex

**Figure 8:**
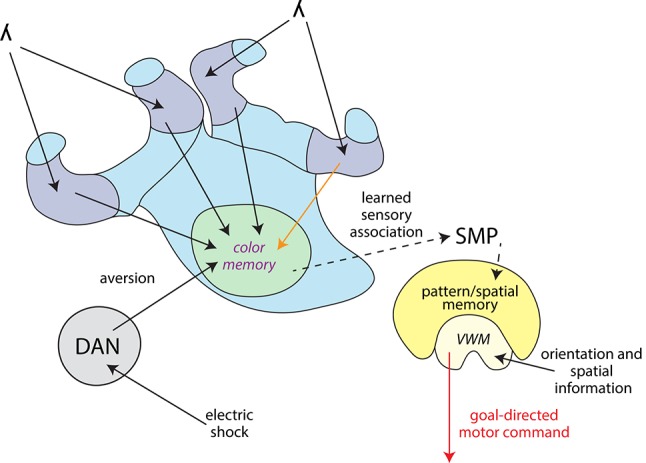
**Information flow model for differential color learning in a binary choice assay**. Information about the light wavelength (λ) enters the collar region (dark blue) of the MBC from the optic neuropils. Visual information is passed on from the collar region to the VL (light green) via Kenyon cells. This process was partially disrupted by a procaine injection into one collar region (orange arrow). Electric shock information is passed on from the ventral nerve cord to dopaminergic neurons (DAN, gray) which modulate MB output. In the VL wavelength information is associated with aversion and most likely color memories are formed here. This process was disrupted by procaine-injections into the VL (marked in purple). Information about the learned sensory association might be passed on indirectly to the CX (yellow) via the superior medial protocerebrum (SMP). The CX receives orientation and spatial information and processes how the animal is orientated in relation to its environment using visual working memory (VWM). The CX initiates a goal-directed motor response, possibly modified in regards to the learned sensory association. This process was disrupted by procaine-injections into the CX (red arrow).

Taken together, we showed that both, the MBs and the CX contributed to the behavioral response to a learned light stimulus. We propose the MBs integrated the coinciding shock and light information and the CX initiated the escape from the light field. We summarized the information flow between the different brain regions with the addition of other findings from different insect orders (Figure 8). To integrate coinciding shock and light information, both stimuli need to be received by the MBs. In the fruit fly γ lobe (part of the VL), a descending Kenyon cell carrying olfactory information forms synapses along the axon with a set of MB output neurons. Dopaminergic neurons modulate these individual compartments in relation to the internal state of the animal (Cohn et al., 2015). In flies, a group of these dopaminergic neurons (PPL1 cluster) carry information of aversive stimuli such as electric shocks (Waddell, 2013; Kaun and Rothenfluh, 2017). It needs to be studied, however, if this process is also found in other insect orders. In fruit flies, olfactory short-term memory is formed in the γ lobes which transitions into long-term memory to α and β lobes via the α' and β' lobes. Kenyon cells which convey wavelength and intensity information to the collar (Vogt et al., 2016) descend into the γ lobes in fruit flies. It remains to be investigated where exactly visual memories relating to color information are formed and where they transition from short-term to long-term memories.

A great question remains, whether there is a connection between the MBs and the CX. A direct connection between the MBs and the CX has not been found so far, with the exception of a single neuron recently discovered in the monarch butterfly (Heinze et al., 2013). An indirect connection could be found in the superior medial protocerebrum (Strausfeld and Hirth, 2013), which comprises outputs from the MBs carrying visual information in fruit flies (Ito et al., 1998) as a well as inputs to the upper division of the central body found in different insects (Strausfeld and Hirth, 2013; Pfeiffer and Homberg, 2014). It is therefore possible that information about the learned sensory association generated by the MBs is passed on indirectly to the CX in order to produce the conditioned response. Evidence for a connection between the MBs and CX manifesting in behavior was found when a sensory preconditioning paradigm involving cross-modal stimuli was investigated (Zhang et al., 2013). Here, an olfactory stimulus and a visual stimulus based on elevation were pre-conditioned. Then one stimulus was paired with reinforcement. A subsequent test of the other stimulus produced a response, even though it was never reinforced. Tested individually, blocking part of the MBs abolished olfactory memory and blocking part of the ellipsoid body (part of the CX in the fruit fly) abolished visual elevation memory. Remarkably, when the olfactory stimulus was reinforced after pre-conditioning and MBs were blocked, animals responded to the visual elevation stimulus. Thus, an association of the two CSs must have occurred in the pre-conditioning.

To explore the connection between the MBs and the CX will be a challenge in the future. The vast knowledge gained about learning and memory in the honey bee field in combination with pharmacological techniques (Felsenberg et al., 2011; Søvik et al., 2016) and assays such as APIS could provide a powerful tool to uncover how the different brain regions interact.

## Author contributions

All authors (JP, BE, NK, US, CG, and AB) contributed substantially to the design and conception of the experiments, analysis and interpretation of the data and revision of the manuscript. JP, BE, and US contributed to acquisition and analysis of the data. JP and NK contributed to R analysis and statistical analysis of the data. JP and BE drafted the manuscript.

### Conflict of interest statement

The authors declare that the research was conducted in the absence of any commercial or financial relationships that could be construed as a potential conflict of interest.
