# Supplementary material for: Different Roles for Honey Bee Mushroom Bodies and Central Complex in Visual Learning of Colored Lights in an Aversive Conditioning Assay
Source: Front Behav Neurosci. 2017 May 30;11:98. doi: 10.3389/fnbeh.2017.00098 (PMC5447682; doi:10.3389/fnbeh.2017.00098)
Supplement: Supplementary file 1 [file DataSheet1.docx]

Supplementary Material


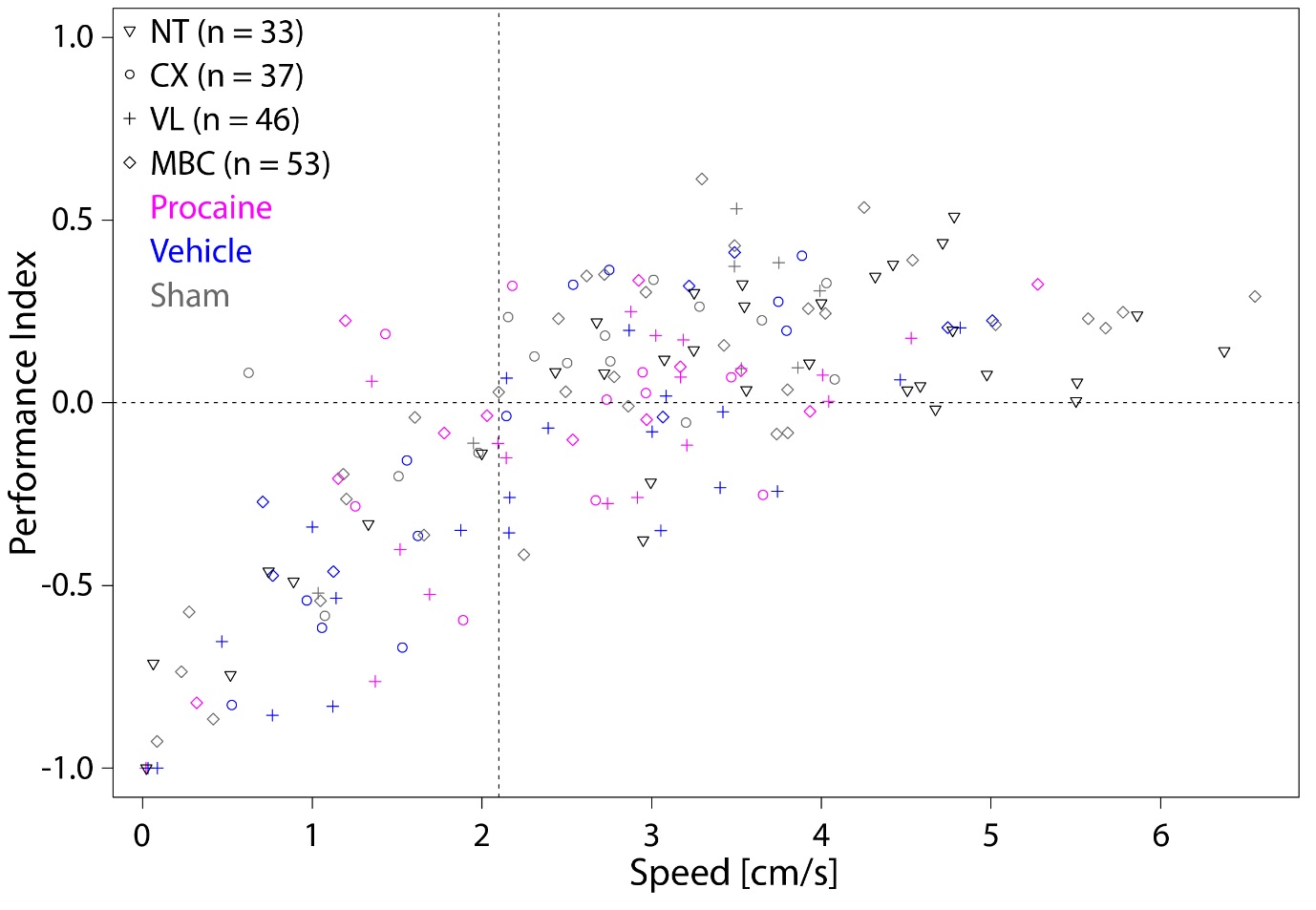


**Figure S1**: Correlation of Performance Index (PI) with speed in the chamber. Mean speed and PIs across all nine training trials and all four test trials are shown for each individual animal. Bees with a mean speed lower than 2.1 cm/s (dashed vertical line) rarely achieve a positive PI (above horizontal dashed line) which indicates learning, and were excluded from further analyses. 7 out of 33 NT animals, 6 out of 12 vehicle animals injected into the CX, 3 out of 10 procaine animals injected into the CX, 4 out of 15 sham animals injected into the CX, 8 out of 21 vehicle animals injected into the VLs, 6 out of 17 procaine animals injected into the VLs, 2 out of 8 sham animals injected into the VLs, 3 out of 8 vehicle animals injected into the MBC, 5 out of 12 procaine animals injected into the MBC, and 10 out of 33 sham animals injected into the MBC were excluded.


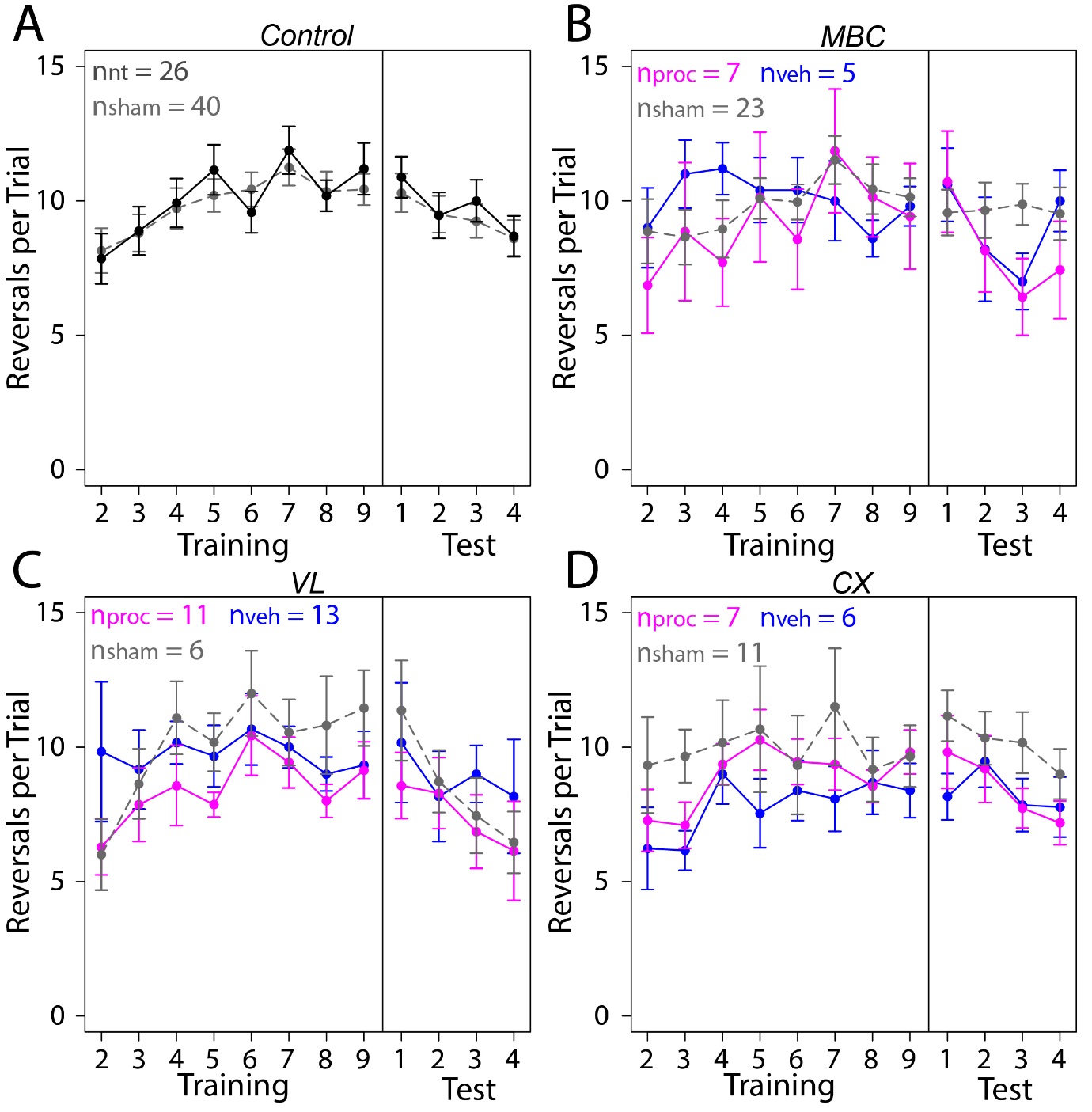


**Figure S2**: Number of Reversals per Trial after one light-shock pairing. NT is shown in black, sham in grey, vehicle in blue and procaine in magenta. Mean number of reversals (± SEM) are shown for each trial for control animals (A), for animals injected into the collar region of the MBC (B), for animals injected into the VLs (C) and for animals injected into the CX (D). LMMs indicated no effects off treatment on Reversals per Trial for any of the four variables (Table S1).


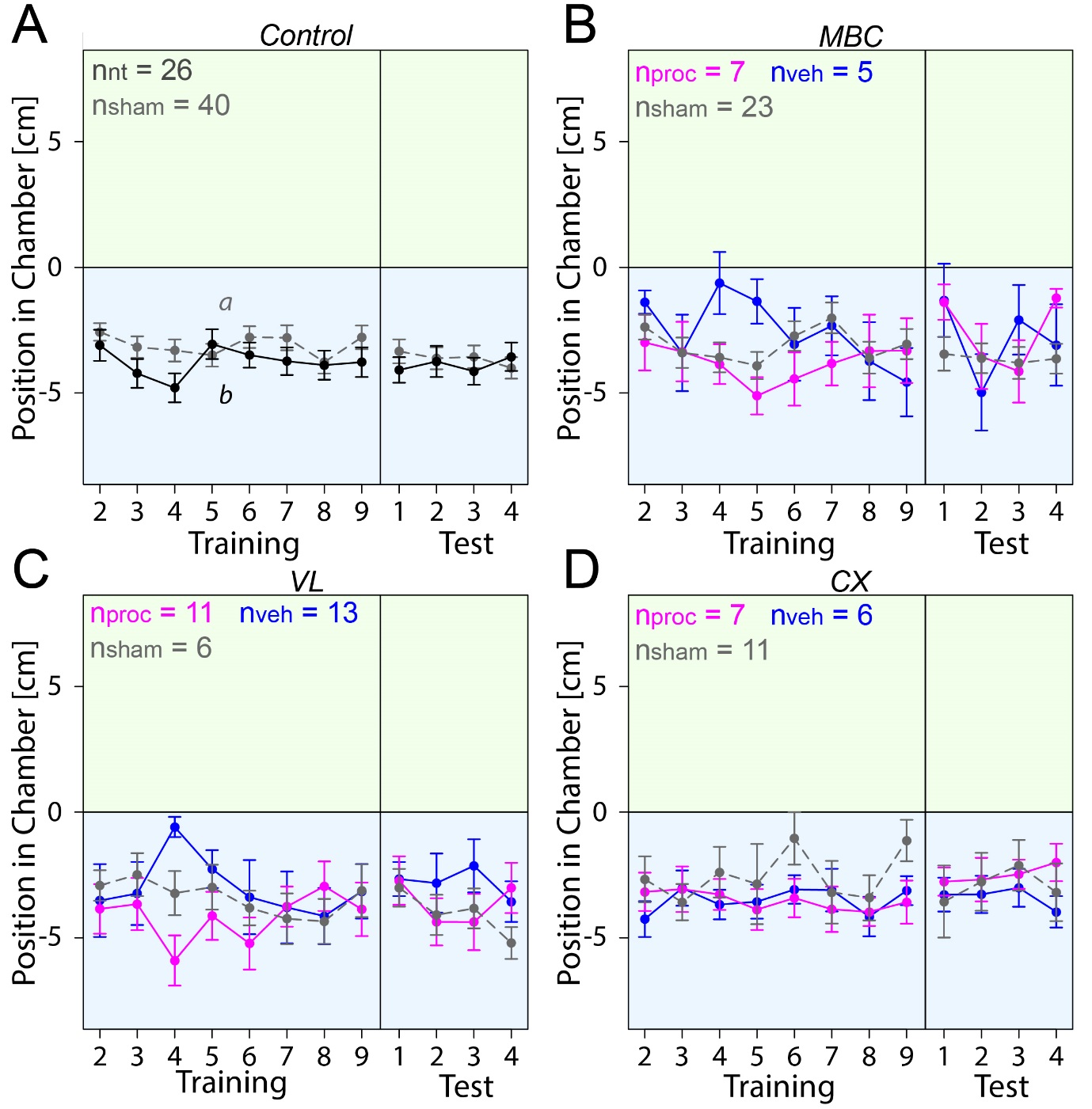


**Figure S3**: Position at light-onset after first trial. NT is shown in black, sham in grey, vehicle in blue and procaine in magenta. Mean position (± SEM) is shown for each trial for control animals (A), for animals injected into the collar region of the MBC (B), for animals injected into the VLs (C) and for animals injected into the CX (D). LMMs indicated no effects of treatment on position for animals injected into the MBC, the VLs or the CX (Table S1). An LMM revealed an effect of treatment on position for the control group (Table S1) as indicated by the letters a and b.

**Table S1:** Summary of Linear Mixed Model (PI, Speed, Reversing Difference, Crossing Latency, Position in Chamber) and Generalized Linear Mixed Model (Reverses per Trial) results for effect of treatment on variable. LMM or GLMM testing sham against NT (Control) and vehicle (veh) or procaine (proc) against sham after injections into the ventral lobes (VL), central complex (CX) or the mushroom body calyx (MBC). Degrees of Freedom (DF); Estimate (Est); Standard Error (SE).

|  |  |  |  | **Training** | | | | **Test** | | | |
| --- | --- | --- | --- | --- | --- | --- | --- | --- | --- | --- | --- |
|  |  |  | DF | Est | SE | t/z | p | Est | SE | t/z | p |
| **Control** | *PI* | sham | 32 | 0.051 | 0.052 | 0.975 | 0.33 | 0.084 | 0.086 | 0.978 | 0.33 |
|  | *Speed* |  |  | -0.616 | 0.282 | -2.188 | 0.03 | -0.290 | 0.265 | -1.097 | 0.28 |
|  | *Reversing Difference* |  |  | 0.330 | 0.624 | 0.529 | 0.60 | 1.253 | 0.890 | 1.407 | 0.16 |
|  | *Crossing Latency* |  |  | 0.086 | 0.409 | 0.210 | 0.83 | 0.148 | 0.649 | 0.227 | 0.82 |
|  | *Reverses per Trial* |  |  | -0.012 | 0.077 | -0.154 | 0.88 | -0.041 | 0.094 | -0.433 | 0.67 |
|  | *Position in Chamber* |  |  | 0.673 | 0.332 | 2.029 | 0.047 | 0.250 | 0.356 | 0.703 | 0.48 |
| **MBC** | *PI* | veh | 32 | 0.042 | 0.108 | 0.392 | 0.70 | -0.014 | 0.168 | -0.082 | 0.94 |
|  |  | proc |  | -0.197 | 0.095 | -2.080 | 0.046 | 0.107 | 0.147 | 0.729 | 0.47 |
|  | *Speed* | veh |  | 0.361 | 0.622 | 0.581 | 0.57 | -0.558 | 0.494 | -1.130 | 0.27 |
|  |  | proc |  | -0.505 | 0.544 | -0.929 | 0.36 | -0.005 | 0.432 | -0.011 | 0.99 |
|  | *Reversing Difference* | veh |  | 0.680 | 1.256 | 0.542 | 0.59 | -1.004 | 1.621 | -0.612 | 0.54 |
|  |  | proc |  | -0.816 | 1.098 | -0.743 | 0.46 | 0.517 | 0.418 | 0.365 | 0.72 |
|  | *Crossing Latency* | veh |  | 0.058 | 0.890 | 0.065 | 0.95 | 1.000 | 1.191 | 0.837 | 0.41 |
|  |  | proc |  | 1.451 | 0.779 | 1.863 | 0.07 | -1.353 | 1.042 | -1.298 | 0.20 |
|  | *Reverses per Trial* | veh |  | 0.050 | 0.141 | 0.354 | 0.72 | -0.056 | 0.177 | -0.314 | 0.75 |
|  |  | proc |  | -0.086 | 0.125 | -0.690 | 0.49 | -0.171 | 0.157 | -1.092 | 0.28 |
|  | *Position in Chamber* | veh |  | 0.524 | 0.594 | 0.882 | 0.38 | 0.760 | 0.770 | 0.987 | 0.33 |
|  |  | proc |  | -0.697 | 0.519 | -1.341 | 0.19 | 1.051 | 0.674 | 1.509 | 1.28 |
| **MBC** |  |  | **Preference Test** | | | | |  |  |  |  |
|  |  |  | DF | Estimate | SE | t | p |  |  |  |  |
|  | *PI* | veh | 32 | 0.698 | 0.265 | 2.631 | 0.01 |  |  |  |  |
|  |  | proc |  | -0.767 | 0.232 | -3.31 | > 0.01 |  |  |  |  |

**Table S1 *continued***

|  |  |  |  | **Training** | | | | **Test** | | | |
| --- | --- | --- | --- | --- | --- | --- | --- | --- | --- | --- | --- |
|  |  |  | DF | Est | SE | t | p | Est | SE | t | p |
| **VL** | *PI* | veh | 27 | -0.414 | 0.098 | -4.217 | < 0.001 | -0.301 | 0.233 | -1.292 | 0.21 |
|  |  | proc |  | -0.266 | 0.101 | -2.638 | 0.01 | -0.329 | 0.240 | -1.375 | 0.18 |
|  | *Speed* | veh |  | -0.624 | 0.397 | -1.571 | 0.13 | -0.398 | 0.528 | -0.754 | 0.46 |
|  |  | proc |  | -0.363 | 0.108 | -0.888 | 0.38 | -0.573 | 0.543 | -1.055 | 0.30 |
|  | *Reversing Difference* | veh |  | -3.396 | 1.093 | -3.107 | < 0.01 | -2.615 | 1.991 | -1.314 | 0.20 |
|  |  | proc |  | -4.009 | 1.124 | -3.567 | < 0.01 | -3.500 | 2.047 | -1.701 | 0.10 |
|  | *Crossing Latency* | veh |  | 2.138 | 1.044 | 2.047 | 0.05 | 0.790 | 1.695 | 0.466 | 0.65 |
|  |  | proc |  | 1.312 | 1.074 | 1.221 | 0.23 | 1.700 | 1.743 | 0.975 | 0.34 |
|  | *Reverses per Trial* | veh |  | -0.259 | 0.144 | -1.800 | 0.07 | -0.226 | 0.154 | -1.467 | 0.14 |
|  |  | proc |  | -0.098 | 0.147 | -0.668 | 0.50 | -0.194 | 0.159 | -1.222 | 0.22 |
|  | *Position in Chamber* | veh |  | -0.963 | 0.663 | -1.453 | 0.16 | -0.475 | 0.666 | -0.713 | 0.48 |
|  |  | proc |  | -0.994 | 0.682 | -1.457 | 0.16 | 0.428 | 0.685 | 0.624 | 0.54 |
| **CX** | *PI* | veh | 21 | 0.086 | 0.090 | 0.953 | 0.35 | 0.062 | 0.229 | 0.272 | 0.79 |
|  |  | proc |  | -0.216 | 0.086 | -2.512 | 0.02 | -0.090 | 0.218 | -0.412 | 0.68 |
|  | *Speed* | veh |  | -0.043 | 0.313 | -0.137 | 0.89 | 0.353 | 0.592 | 0.596 | 0.56 |
|  |  | proc |  | -1.870 | 0.298 | -0.628 | 0.54 | 0.039 | 0.564 | 0.069 | 0.95 |
|  | *Reversing Difference* | veh |  | 0.818 | 1.046 | 0.780 | 0.44 | 0.295 | 2.053 | 0.144 | 0.89 |
|  |  | proc |  | -2.628 | 1.000 | -2.626 | 0.02 | -1.380 | 1.956 | -0.707 | 0.49 |
|  | *Crossing Latency* | veh |  | -0.249 | 0.586 | -0.425 | 0.68 | -0.258 | 1.757 | -0.147 | 0.88 |
|  |  | proc |  | 1.376 | 0.558 | 2.467 | 0.02 | 0.510 | 1.674 | 0.304 | 0.76 |
|  | *Reverses per Trial* | veh |  | -0.009 | 0.127 | -0.071 | 0.94 | 0.042 | 0.219 | 0.192 | 0.85 |
|  |  | proc |  | -0.160 | 0.122 | -1.302 | 0.19 | -0.090 | 0.210 | -0.430 | 0.67 |
|  | *Position in Chamber* | veh |  | 0.384 | 0.829 | 0.463 | 0.65 | 1.124 | 0.696 | 1.780 | 0.09 |
|  |  | proc |  | -0.775 | 0.790 | -0.981 | 0.34 | 0.414 | 0.663 | 0.625 | 0.54 |
